# The Influence of the Material Structure on the Mechanical Properties of Geopolymer Composites Reinforced with Short Fibers Obtained with Additive Technologies

**DOI:** 10.3390/ijms23042023

**Published:** 2022-02-11

**Authors:** Kinga Korniejenko, Pavel Kejzlar, Petr Louda

**Affiliations:** 1 Faculty of Material Engineering and Physics, Cracow University of Technology, 37 Jana Pawła II Street, 31-864 Cracow, Poland; 2 Department of Material Science, Faculty of Mechanical Engineering, Technical University of Liberec, 2 Studenstká Street, 461 17 Liberec, Czech Republic; pavel.kejzlar@tul.cz (P.K.); petr.louda@tul.cz (P.L.)

**Keywords:** geopolymer composite, flax fiber, carbon fiber, 3D printable geopolymer

## Abstract

Additive manufacturing technologies have a lot of potential advantages for construction application, including increasing geometrical construction flexibility, reducing labor costs, and improving efficiency and safety, and they are in line with the sustainable development policy. However, the full exploitation of additive manufacturing technology for ceramic materials is currently limited. A promising solution in these ranges seems to be geopolymers reinforced by short fibers, but their application requires a better understanding of the behavior of this group of materials. The main objective of the article is to investigate the influence of the microstructure of the material on the mechanical properties of the two types of geopolymer composites (flax and carbon-reinforced) and to compare two methods of production of geopolymer composites (casting and 3D printing). As raw material for the matrix, fly ash from the Skawina coal power plant (located at: Skawina, Lesser Poland, Poland) was used. The provided research includes mechanical properties, microstructure investigations with the use of scanning electron microscope (SEM), confocal microscopy, and atomic force microscope (AFM), chemical and mineralogical (XRD-X-ray diffraction, and XRF-X-ray fluorescence), analysis of bonding in the materials (FT-IR), and nuclear magnetic resonance spectroscopy analysis (NMR). The best mechanical properties were reached for the sample made by simulating 3D printing process for the composite reinforced by flax fibers (48.7 MPa for the compressive strength and 9.4 MPa for flexural strength). The FT-IR, XRF and XRD results show similar composition of all investigated materials. NMR confirms the presence of SiO_4_ and AlO_4_ tetrahedrons in a three-dimensional structure that is crucial for geopolymer structure. The microscopy observations show a better coherence of the geopolymer made in additive technology to the reinforcement and equal fiber distribution for all investigated materials. The results show the samples made by the additive technology had comparable, or better, properties with those made by a traditional casting method.

## 1. Introduction

Geopolymers are sometimes called inorganic aluminosilicate polymers and are generally obtained in the reaction of ortosilican polycondensation [1,2]. Common raw materials for the geopolymerization process are metakaolin, calcined clays, industrial waste and by-products (e.g., ash, slag, glass waste, red mud, mine tailings, for example: copper, vanadium), gauges, etc. or other natural and artificial silicoaluminates (e.g., zeolite, pure Al_2_O_3_–2SiO_2_ powder, as well as minerals containing magnesium [2,3,4]. Nowadays, geopolymers are not only being researched, but they have also has been used in real-life civil engineering applications. The most spectacular examples are the Global Change Institute (GCI) building at the University of Queensland in Australia and the Wellcamp airport in Brisbane, Australia [5,6]. However, these materials are also used in the production of construction materials such as the fire-resistant wood panels, sandwich panels and other building elements, heat shields for space shuttles, as well as fire barriers in the construction industry, protective coating in emergency repair runways, material for the support to the stabilization of toxic waste, including radioactive substances and other applications [7,8,9,10].

Today, interest in the practical application of geopolymers is increasing due to a growing public awareness of the need to protect the environment. Geopolymer composites appear to be the most promising environmentally friendly alternative to traditional cementitious materials, including Portland cement [1,11]. Production of geopolymers results in a much lower carbon footprint than traditional construction materials [2,12]. It allows one to reduce the emission of CO_2_ and other substances harmful to the environment and at the same time save natural resources by using waste [4,13]. Additionally, the geopolymerization process allows different wastes streams to be used as a raw materials [4,14]. The surplus environmental benefits could be achieved by using the appropriate production technology, such as additive manufacturing [15,16,17].

Now, the technology of additive manufacturing develops rapidly in the construction industry [17,18]. It provides new horizons in this sector, especially in terms of geometric flexibility, reduction in labor costs, improvement of efficiency and safety, construction in harsh environments, and sustainability [19,20]. The important element is also the improvement of efficiency and safety, as well as the reduction in costs, especially related to manpower [17]. The additive technology also reduces waste, including waste related to formwork construction [15,17]. Not only is a smaller amount of material used for molding and casting operations, but also the technology offers the possibility of optimizing the construction, and it finally reduces the amount of material used during the process. An extra benefit is a reduction in the cost of transportation through the possibility of the production “in place” and using local waste materials [15,21]. However, the full exploitation of additive technology for effective application still requires optimization, especially with respect to improving methods of designing new materials [17,22].

The ceramic materials dedicated to additive technology must have a good combination of all essential material properties dedicated to traditional technology such as: proper durability, ductility, vapor imperviousness, high tensile and compressive strength, low coefficient of thermal expansion, resistance to UV light and others [18,20], as well as properties connected with additive technologies, especially short time of curing, proper viscosity and time of bounding [18,23]. The properties most often cited in the literature are as follows:

(1) Pumpability—the reliability of the material that is moved through the delivery system [18];
(2) Printability or extrudability—depositing material through a deposition device or defined as state to pass or be pumped through a small nozzle [18,20];
(3) Buildability—resistance of deposited wet material to deformation under loads or defined as holding the shape under the weight of subsequently printed layers [18,20];
(4) Interlayer bonding and segregation prevention [20];
(5) Open time—period while the aforementioned properties remain consistently within acceptable tolerances [18,20] and sufficient viscosity to retain its shape after the printing process [19,24];
(6) Thixotropic properties (high yield strength and low viscosity behavior) [19];
(7) Proper post-processing procedures [25,26].

Another significant problem is reinforcing additive technology; steel bars, well known from traditional concrete technology, must be replaced by short or long ones. In the case of ceramic materials such as geopolymers, reinforcement is highly required, due to their limited brittle behavior [2,20]. However, it also requires modern technological solutions, including the proper design of composition and modifications in the manufacturing process [27,28,29]. Until now, only a few studies have been conducted in the area of fiber-reinforced composites made in additive technologies [2]. They were focused on:

(1) Long steel fibers [30,31,32],
(2) Short steel fibers [33],
(3) Short glass fibers [34],
(4) Short carbon and flax fibers [16],
(5) Different kinds of plastic fibers, including polypropylene fibers (PP), polyvinyl fibers (PVA) and polybenzoxazole fibers (PBO) [33,35,36,37].

The most important works on this topic are summarized in Table 1.

The provided analysis of the literature shows that reinforcement by short and long fibers is an effective way of reinforcing geopolymers dedicated to additive manufacturing technology, and it also provides a positive influence on flexural strength [17,43]. The most important benefits associated with using fibers as a reinforcement are as follows:

(1) Increasing flexural strength—for samples with fiber addition, it could be even 600% higher than for plain sample [30].
(2) Improving interlayer bonding, including influence of time intervals between layers-reducing time gaps between additive layers and introducing fibers impact on the bond strength between subsequent layers [35].
(3) Laminating the cracking propagation and reducing brittle behavior of the geopolymers [16].
(4) Reducing shrinkage [40].

The main objective of the article is to investigate the influence of material microstructure on the mechanical properties of the two types of geopolymer composites (flax and carbon-reinforced) and to compare two methods of production for geopolymer composites (casting and 3D printing). These two types of fibers were selected to compare synthetic and natural ones. Carbon fiber was selected because it has the best mechanical properties and was previously applied in the geopolymer matrix [16,32]. This fiber has properties better than those of popularly used glass fibers and is resistant to alkali environment in geopolymer matrix. The main limitation in the wider application of this fiber is the price, which is much higher than in the case of glass or polymer fibers. The flax fiber was selected as an alternative to synthetic fibers. In central Europe (Poland and the Czech Republic), this fiber is one of the two plants of naturally growing fibers (flax and hemp). It is also waste product from the connected plant production. The selection of flax fibers was based on previous research in which these two natural fibers were compared.

## 2. Results

### 2.1. Mechanical Properties–Compressive and Flexural Strength

**Figure 1:**
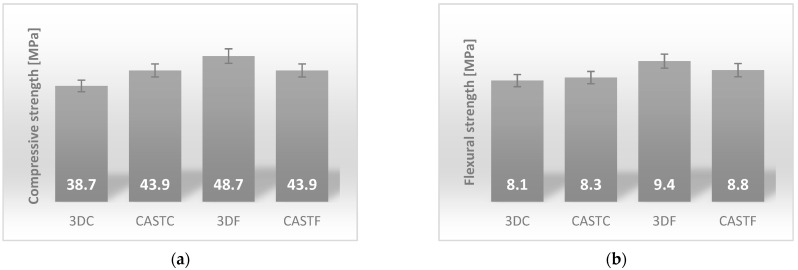
Mechanical properties: (**a**) compressive strength; (**b**) flexural strength.

The samples were investigated with regard to their mechanical properties after 28 days [16,44]. A summary of these results is presented in Figure 1 [44].

The best value of the compressive strength was reached for the sample made by simulating a 3D printing process for the composite reinforced by flax fibers. It was about 48.7 MPa. Both of the casted samples were approximately 43.9 MPa. Geopolymer composites with the addition of 1% by weight of carbon fibers made by simulating the 3D printing process had the lowest compressive strength value, which was approximately 38.7 MPa (Figure 1).

In the case of flexural strength, the values of the composite with flax fibers were significantly better than those of the reinforced carbon fibers. The best value of the flexural strength was reached again for the sample made by simulating the 3D printing process for the composite reinforced with flax fibers. It was about 9.4 MPa. The casted sample reinforced with flax fiber achieved a value of 8.8 MPa. The results for the samples reinforced by carbon fibers were 8.3 MPa for the casted sample and 8.1 MPa for the sample made by simulating the 3D printing process, respectively (Figure 1). The unexpected finding in this research was the very high values of compressive and flexural strength of the composite with flax fibers in comparison to the composite with carbon fibers [16,44]. Taking into consideration this fiber’s properties, the results should be different, because the carbon fibers have better mechanical properties than flax fibers.

### 2.2. Analysis of Bonding in the Material with the Use of Fourier-Transform Infrared Spectroscopy (FT-IR)

**Figure 2:**
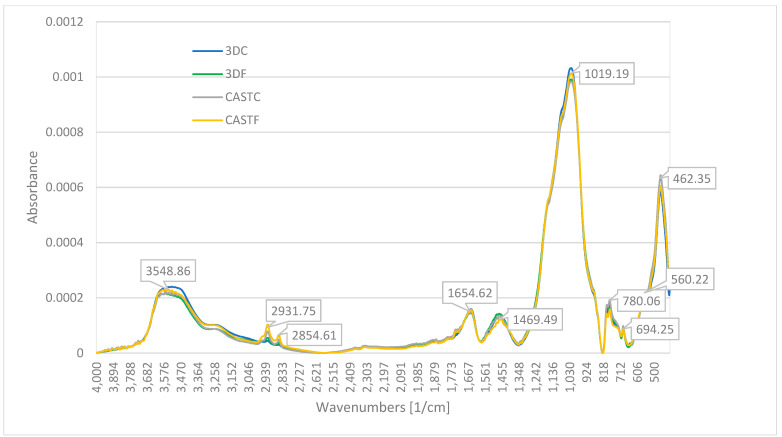
FT-IR for geopolymer composites.

Figure 2 shows the FT-IR spectra for the geopolymer composites. The patterns for all compositions are quite similar. There is a lack of significant differences between particular composites and a lack of differences between materials manufactured using different methods.

FT-IR spectra are typical for geopolymer materials and shows bonds typical for their internal structure [5]. The peak at a wave number of 3548 cm^−1^ represents the stretching vibration of the free O–H group. Similarly, the peak at 1640 cm^−1^ represents the bending vibration of hydrogen-bonded O–H group (absorbed water) and the peak at wavenumbers of 2931 represents the stretching vibration of hydrogen-bonded O–H group [5,45,46]. It could indicate a small amount of molecular water. The peak at 2854 cm^−1^ could represent C–H bond [4,47].

The peaks at a wave number of 1469 characterize the stretching vibration of the C–O bond, which could be related to sodium bicarbonate-Na_2_CO_3_ [48,49].

The broad and strongest peak at a wavenumber of 1019 cm^−1^ is the asymmetric stretching vibration of the Si–O–T bond (where T denotes Si or Al) [5,50,51,52]. It is probably associated with asymmetric stretching vibrations and also with Si–O(Si). The peak at about 460 cm^−1^ connected with bending vibrations Si-O(Si) presented in silicate tetrahedra [52,53]. The peaks at 780 and 694 cm^−1^ represent the crystalline phase of quartz components [6,52,53].

The analysis shows that all geopolymer composites have a similar internal structure, consisting of free O-H, hydrogen bonded O–H, C–O, Si–O–Al and Si–O–Si as well as the crystalline phase of quartz (Table 2). FT-IR clearly shows the presence of aluminum silicates and/or aluminosilicates in geopolymers.

### 2.3. Chemical and Mineralogical Composition with the Use Spectroscopy (XRD-X-ray Diffraction and XRF-X-ray Fluorescence)

The elemental and oxide compositions investigated by XRF are presented in Table 3 and Table 4. They have been compared with the composition of main raw materials—sand and fly ash.

As expected, the main elements of the composite structure are: oxygen (O), silica (Si), and aluminum (Al). These elements come from raw materials used and are the basic element for creating the structure of the geopolymer. The reinforcement and the method of the production do not have any significant influence on the elemental characterization. All compositions include sodium (Na), which comes from the sodium promoter that is used during the manufacturing process. The analysis also show the small amount of iron (Fe) and calcium (Ca) in the material (Table 3).

The oxide composition, presented in Table 4, is essential for geopolymerization process.

The main oxides presented in the structure are: alumina-Al_2_O_3_ and silica-SiO_2_. This is typical for geopolymer materials. Moreover, the composite includes significant amounts of Fe_2_O_3_, Na_2_O and CaO. The obtained data are coherent with the applied raw materials; in particular, they confirmed the applied fly ash has a composition relevant for the class F. This class is characterized by a high percentage of silica (SiO_2_), alumina (Al_2_O_3_), and iron oxide (Fe_2_O_3_)–min. 70% and, in addition, low percentage of calcium oxide (CaO)–max. 4% [54,55]. The compositions achieved mixtures according to their oxide composition are very similar. There is no significant differences between compositions and method of production. The alumina-Al_2_O_3_ is in range up 18.207 to 18.537% and the silica-SiO_2_ between 54.453 and 57.003%. The weight ratios crucial oxides such as: SiO_2_/Al_2_O_3_, CaO/Al_2_O_3_, CaO/SiO_2_, SiO_2_/Na_2_O and Al_2_O_3_/Na_2_O are suitable for geopolimerization and are similar for all compositions analyzed [56,57]. Additionally, the low amount of CaO is positive for a geopolymerization. It supports a longer time taken for material bounding thanks to the creation of a 3D structure. This kind of structure gives the geopolymers a suitable resistance to environmental conditions [54,58,59]. The advantages can also be seen with some amount of content iron oxide (Fe_2_O_3_). Some research shows that it should positively influence 3D structure [54,55,60] and could be important in high temperature applications [61].

**Figure 3:**
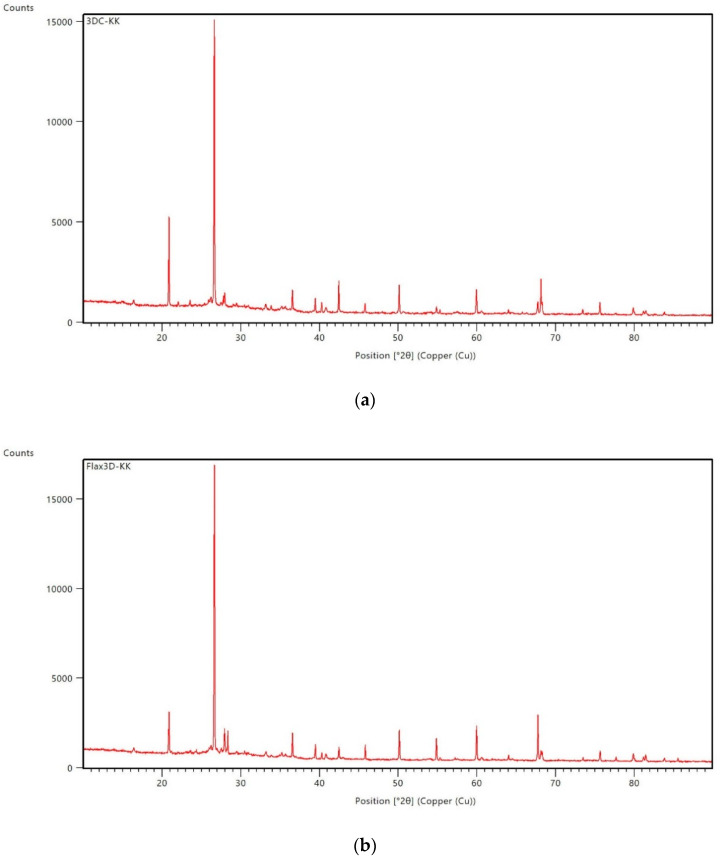

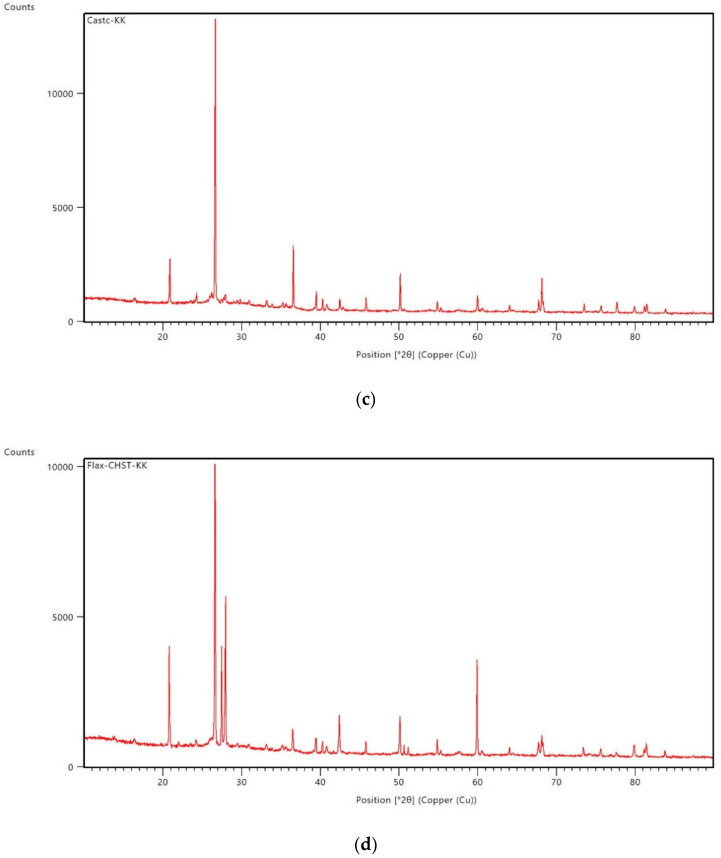
The results of XRD analysis for samples: (**a**) 3DC; (**b**) 3DF; (**c**) CASTC; (**d**) CASTF.

The investigation also involved the determination of the mineralogical structure by XRD. All compositions have a similar structure (Figure 3).

Identified phases are quartz, mullite, hematite, magnetite, anhydrite, albite, and sylimanite. The first two large peaks came from quartz. A significant amount of quartz is associated with the use of sand as an aggregate. The third and fourth peaks, which are slightly visible at Figure 3d, came from albite and anhydrite. These minerals are also popular in the mineral composition of geopolymers [62,63]. The peaks at about 60 are connected with the presence of mullite. The amount of this mineral is slightly different with all of the samples presented. The double peak before 70 is connected to quartz. The other peaks are also connected with the minerals or hematite presented (peak around 43). The mineralogical composition is slightly different for the presented samples, but the variations in results are mainly connected with the amounts of particular minerals in the composition. There should not be a significant influence on the material properties. Among them, there are crystalline as well as amorphous phases. The typical crystalline phases for geopolymer composites are quartz (related to silicon dioxide), mullite, hematite, albite, and anhydrite [54,62]. The curves also indicate the presence of an amorphous phase (indicated by diffuse halo/the curves have a broad hump) [62,63].

### 2.4. Nuclear Magnetic Resonance Spectroscopy (NMR)

To confirm the typical internal structure for geopolymers and find potential differences between compositions, NMR analysis has also been provided. In contrast to geopolymers, the alkaline activated materials do not form 3D networks, but only a 2D structure. It affects the material properties, including different physicochemical and functional properties, such as resistance to chemical agents, functional properties such as fire resistance, and long-term properties, durability [54,64]. Wherein, the mechanical properties of alkaline activated materials could be even higher than that of geopolymers, especially in the short term [64]. At the same time, their resistance and long-term properties are usually worse [64]. The differences are usually visible in NMR microstructural studies [65,66].

For geopolymer materials, the presence of SiO_4_ and AlO_4_ tetrahedrons in a three-dimensional structure is crucial. The reactive aluminosilicates are dissolved, and next in the polycondensation process, the tetrahedrical structures [SiO_4_]_4-_, [AlO_4_]_5-_ combine together with the corners, forming amorphous or sub-crystalline spatial aluminosilicate structures [54,64]. To confirm this structure, an investigation of ^27^Al and ^29^Si was performed for all compositions [66,67].

**Figure 4:**
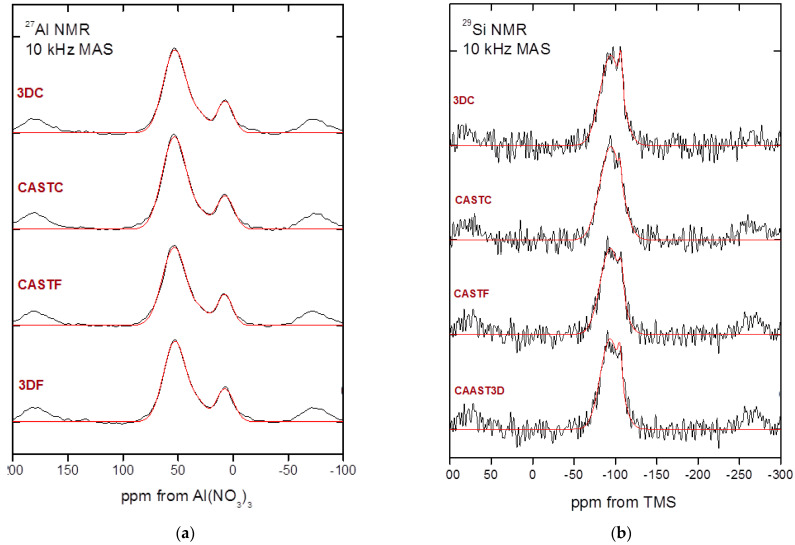
The results of NMR analysis for: (**a**) aluminum; (**b**) silica.

Figure 4 shows the results for all compositions produced by two different methods. There are no significant differences between the samples analyzed.

Aluminate anions, four-coordinated aluminum (with respect to oxygen) resonate at 60–80 ppm, and that in silico-aluminates, four-coordinated aluminum resonates at approximately 50 ± 20 ppm while six-coordinated aluminum resonates at about 0 ± 10 ppm from [Al(H_2_O)_6_]^3+^ [66,67]. The ^27^Al chemical changes, presented in Figure 4 and Table 5, in the range of ca. 55 ppm from [Al(H_2_O)_6_]^3+^ indicate that the aluminum is of the AlQ4(4Si) type and is tetrahedrally coordinated. The ^29^Si MAS-NMR is represented by two peaks (Figure 4). The major band at ca. −94 ppm, is connected with the presence of a SiQ4 (2Al) unit and the second band at −115 ppm is related to unreacted silica-fume-SiO_2_ (Table 5). The second component is probably the result of sand addition [66,67].

The presented data show that all composites have a similar internal structure typical for geopolymer material.

### 2.5. Atomic Force Microscope (AFM)

**Figure 5:**
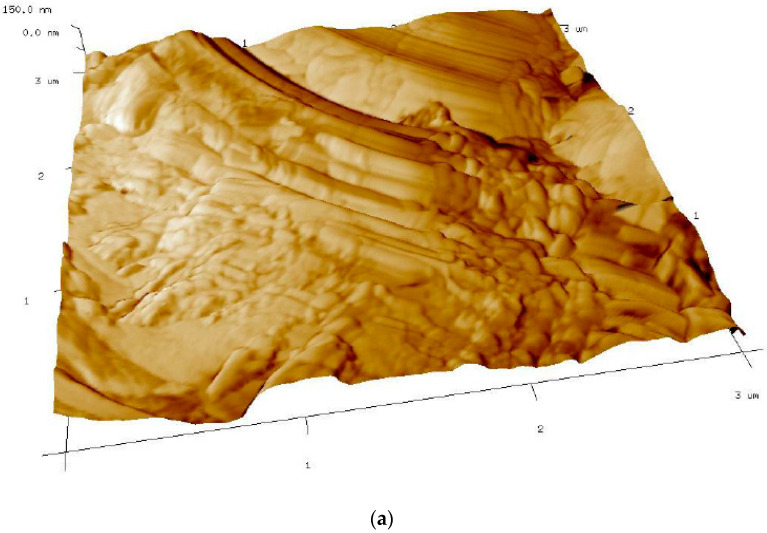

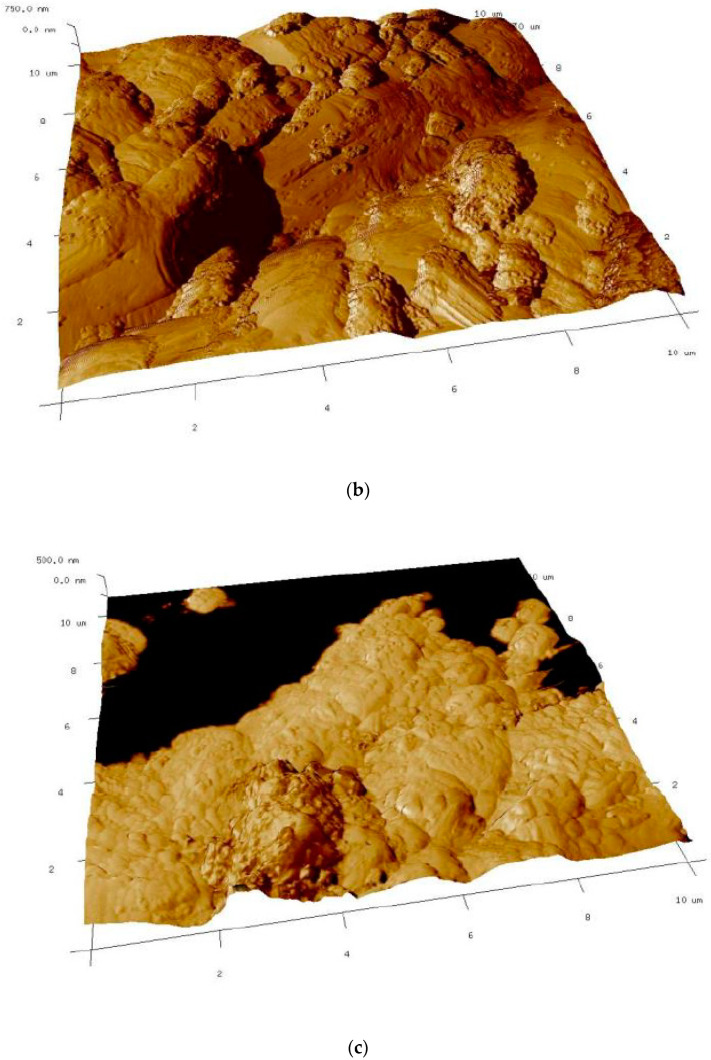

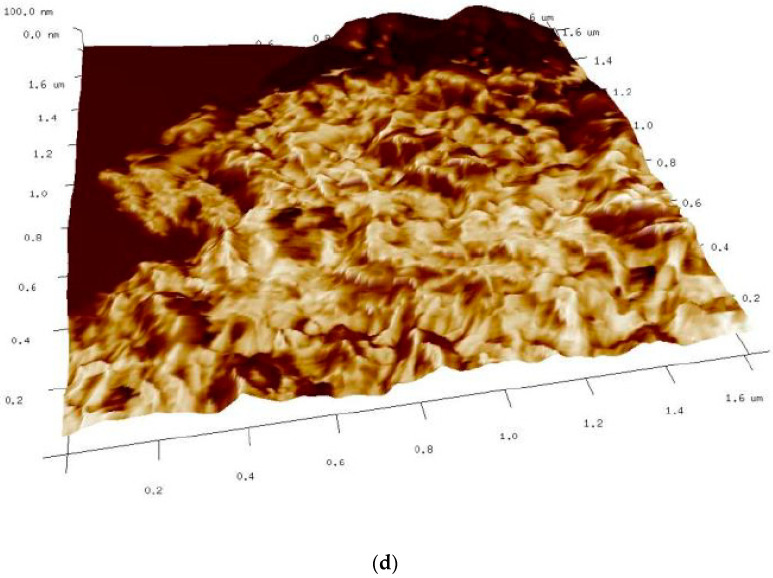
Phase contrast imaging of fiber surface topography in AMF: (**a**) 3DC; (**b**) 3DF; (**c**) CASTC; (**d**) CASTF.

Figure 5 shows the imaging of surface fragments selected for research by atomic force microscopy (AFM) in the semi-contact mode (Tapping Mode) for four samples of geopolymer composites.

The parts of the fibers are visible as a dark-colored phase, and the light-colored phase is a geopolymer material that covers the fiber. The observations show that the CASTC sample has large surfaces of fibers without geopolymer particles. The CASTF sample has small surfaces of the fibers, which are without any geopolymer particles. In the other two samples, 3DC and 3DF, only the fibers covered with geopolymer particles are visible. The entire surface of the fibers is covered; in the phase image of these composites, there are no differences in the phase shift on the surface of the samples. A similar effect may be due to the presence of a different type of fiber in the composite than carbon fibers.

The AMF seems to show better coherence of the geopolymer made in additive technology to the reinforcement. The surface of the fibers is more evenly covered than in casted samples. However, this kind of test is provided on very small samples, and they have a qualitative nature. The results obtained do not explain the differences in mechanical properties between the composites.

### 2.6. Confocal Microscopy

**Figure 6:**
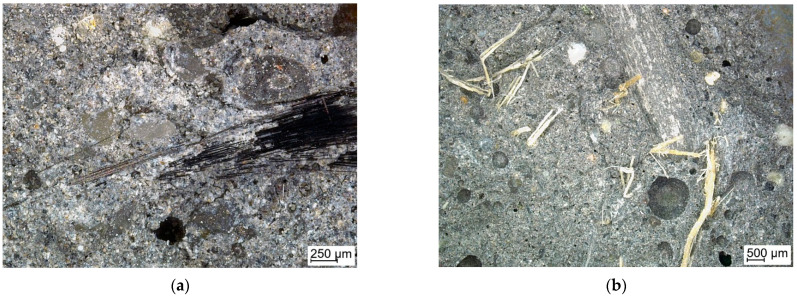
Structure of the composites reinforced with: (**a**) carbon fiber; (**b**) flax fiber.

Observations on the breakthroughs of the samples were provided. They allow the microscopy pictures to be obtained, as well as the information about the surface in larger scale than AMF. Thanks to confocal microscopy, is was possible to observe the material structure in the scale between nano-dimensions (AMF) milliammeter scale (observations with the naked eye). These observations cover the investigation of the surface on a micrometer scale. The confocal microscopy investigation was supplementary to scanning microscopy and allows the 2D structure to be received on the surface topology. This observations were carried out in a smaller magnification in comparison to the presented SEM images for better visibility of fibers distribution. Microscopy observations show the differences between the fiber distributions (Figure 6). The carbon fibers, independent of the production method, have a larger tendency to create the agglomerations.

**Figure 7:**
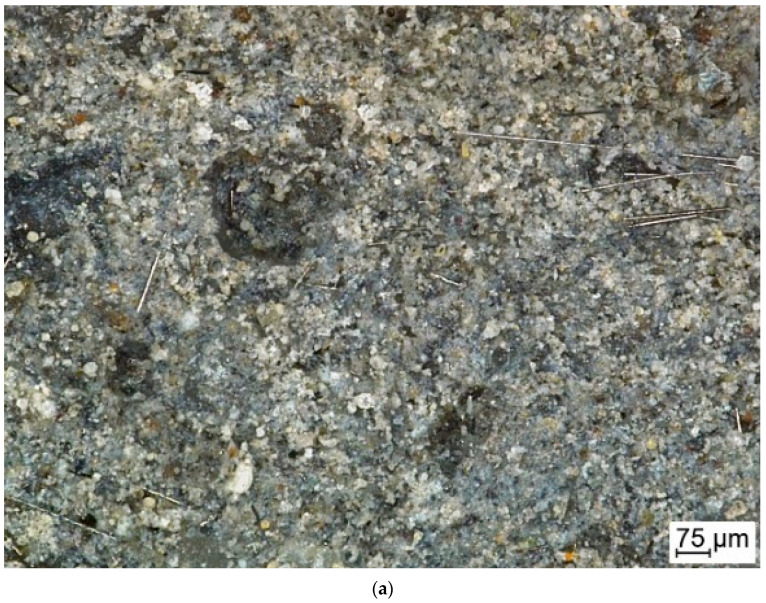

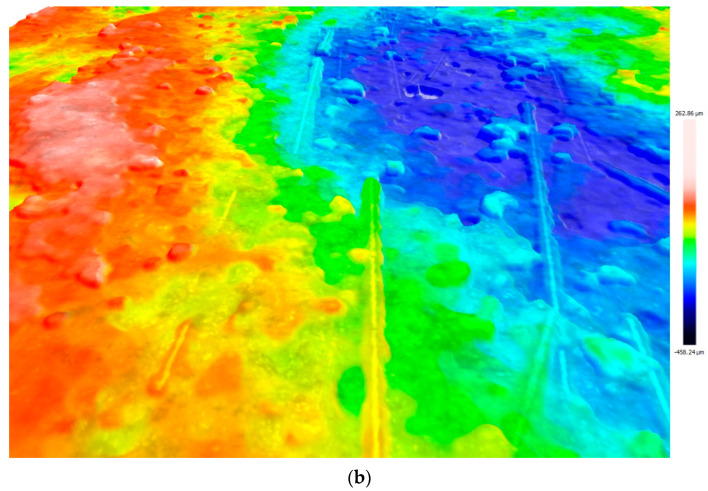
The structure of the carbon fiber reinforced composites: (**a**) structure of the composites and (**b**) surface profile.

Figure 7 presents the surface of the breakthrough of the composite with the surface profile. The distribution the fibers in the figure is even in this fragment. The fibers are clearly visible on the surface of the material (Figure 7b).

**Figure 8:**
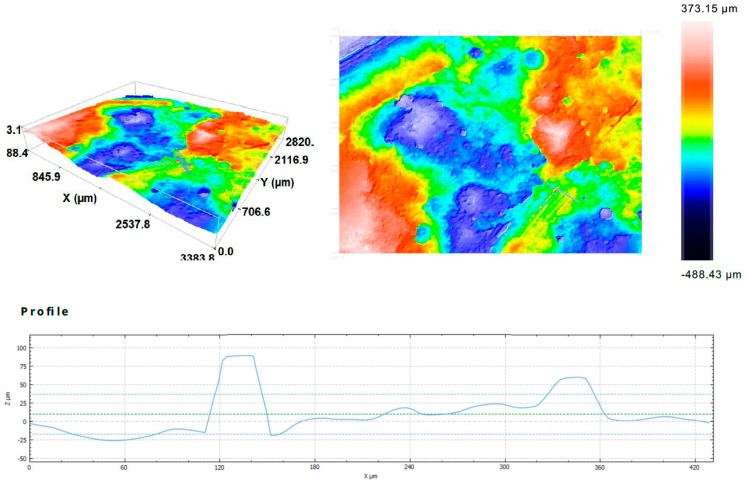
3D confocal microscopy images and profile curve of the carbon fiber reinforced composite made by simulation of additive technology.

Additionally, the surface profile was measured for the chosen area. Figure 8 shows the carbon fiber-reinforced surface profile of the carbon fiber-reinforced composite made by simulation of additive technology. In the profile curve, there are two peaks that give information about the height difference between the surface and the fibers. It also allows one to compare the relatively small width of the fiber with its height above the surface of the composite in the profile curve.

**Figure 9:**
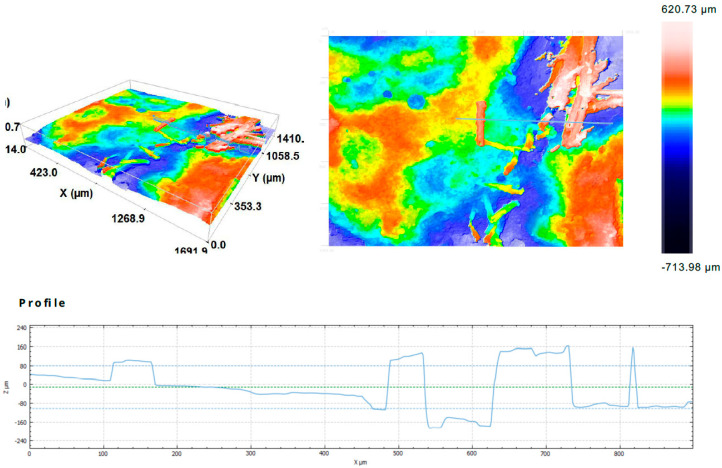
3D confocal microscopy images and profile curve of the reinforced composite with flax fiber and made by simulation of additive technology.

Figure 9 presents the surface profile of the composite reinforced with flax fiber and made by simulation of additive technology. The profile curve has a slightly different character than in the case of carbon fiber. It is rougher. The single fiber is also larger in size than the single carbon fiber, and the height difference is greater (Figure 9).

The observations by confocal microscopy did not reveal significant differences between the samples produced by different methods.

### 2.7. Microstructure Investigations with the Use of Scanning Electron Microscope (SEM)

**Figure 10:**
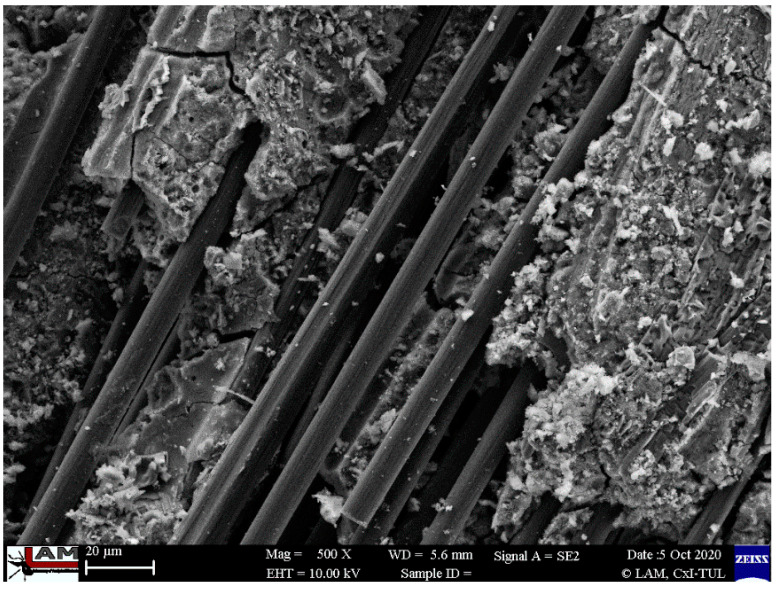
Microstructure of the cast samples reinforced with carbon fiber with visible fibers agglomerations.

**Figure 11:**
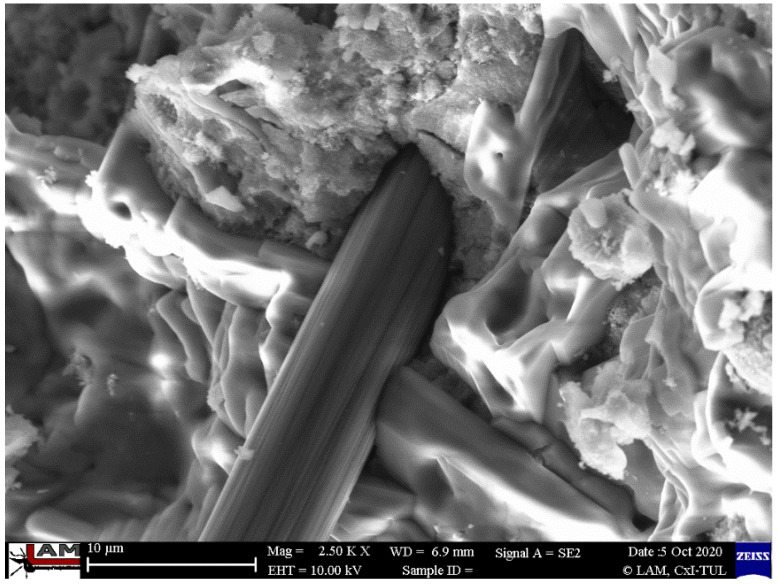
Microstructure of injected samples reinforced with a carbon fibers with visible lack of cohesion between the sample and the matrix.

**Figure 12:**
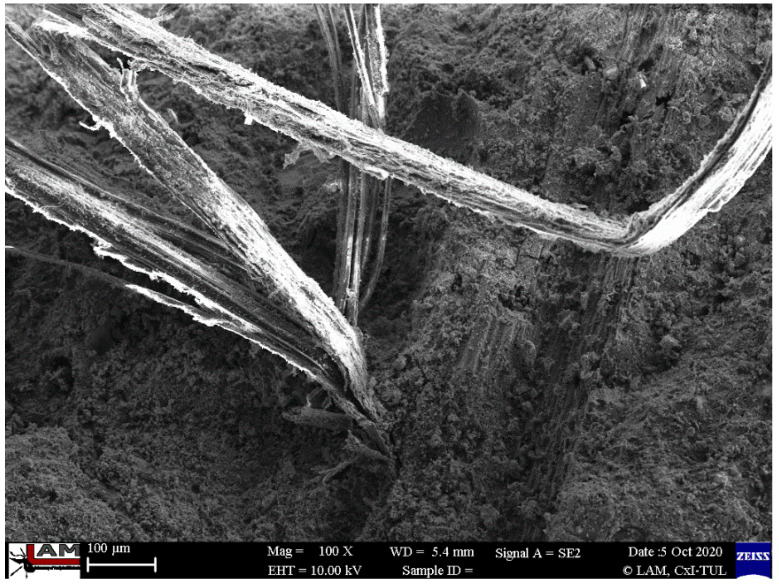
Microstructure of injected samples reinforced with flax fibers.

**Figure 13:**
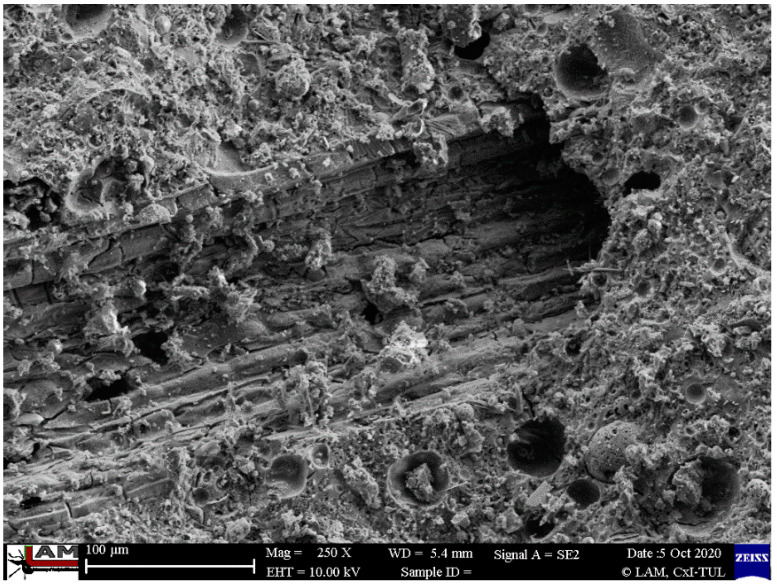
Microstructure of the cast samples reinforced with flax fibers with very good visible cohesion between the sample and the matrix.

**Figure 14:**
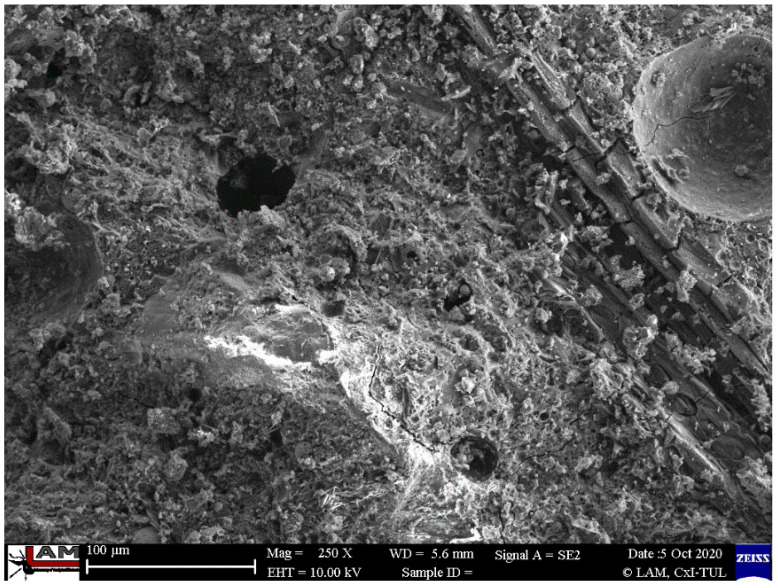
Microstructure of the cast samples reinforced with flax fibers with visible matrix structure and very good cohesion between sample and matrix.

Scanning electron microscope (SEM) research was performed for all composites at different magnifications for the fiber-reinforced composites and for the different methods of production. Figure 10, Figure 11, Figure 12, Figure 13 and Figure 14 show the microstructure of the samples reinforced with carbon and flax fibers. There is lack of differences between the casted samples and those made by simulation of additive technology. There is also a lack of significant differences in all samples in the microstructure of the matrix material.

In Figure 10 and Figure 11, the microstructure of the material reinforced with carbon fibers is depicted.

Figure 10 shows the microstructure where the agglomeration of the carbon fibers is presented. The agglomeration of carbon fibers were previously reported in the literature [44]. It is a consequence of the form of the fibers that are delivered in the form of “small flakes” that are an agglomeration of single fibers. Despite the fact that the flakes are evenly distributed in the volume of the material, there are agglomerations of single fibers in it. This could have a negative influence on the mechanical properties [44,68,69].

The investigation does not confirm the cohesion of the carbon fibers and the geopolymer matrix (Figure 11). The void between the fiber and the matrix is visible. This is probably the main reason that the composites with carbon fibers have worse mechanical properties than those reinforced with flax fiber.

In Figure 12, Figure 13 and Figure 14, the microstructure of the material reinforced with flax fibers is presented. Natural reinforcement is generally reported to have worse mechanical properties than using artificial fibers such as carbon fiber [2,17,70]. Many publications reported the main cause to be the lack of coherence between the fiber and matrix [71,72]. The microstructural investigation does not confirm it in the case of provided research.

Figure 12 shows the structure of the flax fiber in the matrix. The structure is typical of natural fiber, irregular due to its different dimensions and rough surface [44,71].

Figure 13 presents the microstructure of the casted samples reinforced with flax fibers. The external part of the fibers is tightly joined to the matrix. During the mechanical tests, the fibers were broken, but they do not lose the coherence with the matrix.

In Figure 14, the microstructure of the injected samples reinforced with flax fibers is presented. There is no significant difference between the microstructure of the samples with the flax fibers that were injected (simulation of additive technology) and those that were casted. There is very good cohesion between fiber and the visible matrix.

The structure of the matrix is similar to that of the carbon fiber composites. Flax composites are characterized by better coherence between the fiber and the matrix. This partly explains the better mechanical properties of the samples with flax fiber. The microstructure observation does not explain the better results of compressive and flexural strength for the samples prepared by the additive technology method simulation (difference between production methods).

## 3. Discussion

Geopolymer composites based on fly ash reinforced with flax and carbon fibers were produced using two methods: casting, and simulation of additive manufacturing technology simulated by injection molding. In the first step, the mechanical properties were determined. The values obtained for the compressive strength were between 38.7 and 48.7 MPa. The obtained values for the flexural strength were between 8.1 and 9.4 MPa. Both of these results are in the range of values for fiber reinforced geopolymer composites [2,3,72]. The best values were achieved for the composite reinforced with 1% flax fiber made by simulation of the additive manufacturing method, and the worst values were achieved for the composite with 1% carbon fiber made by the same technology. There is a lack of visible tendency to change the mechanical properties of materials depending on the manufacturing method used. The samples reinforced by flax fibers have better mechanical properties than composites reinforced by carbon fiber. This result is not in line with previously published investigations that reported the predominantly better properties of synthetic fibers [1,73].

The investigation presented in the article attempts to explain the influence of material structure on the mechanical properties of the two kinds of geopolymer composites (flax and carbon reinforced) and the comparison of two methods of production for geopolymer composites (casting and 3D printing).

Analysis of bonding in the material by FT-IR shows similar bonds in all types of investigated composites. They consist mainly of free O-H, hydrogen-bonded O-H, C-O, Si-O-T and crystalline phase of quartz. FT-IR clearly shows the presence of aluminum silicates and/or aluminosilicates in geopolymers. FT-IR spectra are typical for geopolymer materials and show bonds typical for their internal structure [5].

The chemical and mineralogical composition (XRD and XRF) also do not show significant differences between the composites. The XRF investigation confirms the usefulness of the raw materials applied for the geopolymerization process. The high aluminum content and low calcium content promote the formation of 3D network in geopolymer materials. Additionally, the low amount of CaO supports the longer bonding process. Too high of a CaO content means that the binding process is too fast and therefore the geopolymerization process is not performed properly [59,74]. It has additional meaning in the case of additive manufacturing technology, where a hardening process that is too fast could have a negative influence on creating a proper interlayer bonding [35,75].

Nuclear magnetic resonance spectroscopy shows that the composites have similar internal structures typical for geopolymer material. The data received are consistent with the findings of other research teams [66,67]. The 3D structure has been effectively created in the material.

The AFM investigation shows the difference between covering the fibers with the geopolymer matrix. It seems to confirm better coherence of the geopolymer made in additive technology with the reinforcement than that produced by the traditional casting method. However, it must be taken into consideration than this kind of test have a qualitative nature. The obtained results do not fully explain the differences in mechanical properties between the composites. There is a lack of comparative works in this area in the literature.

The microstructure was investigated by using confocal microscope as well as scanning electron microscope. The investigations provided by confocal microscopy revealed no significant differences between the samples produced by different methods. SEM observations confirm that the structure of the matrix is typical for fly ash-based geopolymers with aggregate [44] and there is a lack of differences between the matrixes in the four compositions. The investigations also show that the composites with flax fiber are characterized with better coherence between the fiber and matrix. It is extrusion technique.

The structure of the matrix is similar to that of carbon fibers. Flax composites are characterized with better coherence between the fiber and matrix. This partly explains the better mechanical properties of the samples with flax fiber. The microstructure observation does not explain the better results of compressive and flexural strength for the samples prepared by the additive technology simulation method (difference between production methods). This fact is not in line with previously published research, where artificial fibers usually have better coherence than natural ones [2,17,70]. The microscopy analysis does not satisfactorily explain the underlying reasons for the observed differences in the mechanical properties of the composites; in particular, it does not explain the better results of compressive and flexural strength for the samples prepared by simulation of additive technology method simulation (difference between production methods).

The provided research does not show differences between casted samples and samples made by simulation of additive manufacturing technology. These research studies could show the potential for the development of additive manufacturing technology and the potential to replace it in many applications, including the building industry [76,77,78].

## 4. Materials and Methods

### 4.1. Materials

The raw materials for the geopolymer matrix were fly ash and sand ratio of 1:1. The sand was fine-grained saturated-surface dry construction sand (the surfaces of the sand particles are “dry” but the voids between the particles are saturated with water—there is no surface absorption) [14]. The fly ash came from the Skawina Combined Heat and Power Plant (Krakow, Lesser Poland region, Poland). It has a typical chemical composition for class F, contains up to 5% unburned material, less than 10% iron compounds, and a low amount of calcium compounds [44,54,79]. Fly ash has good reactivity and workability: the reactive silica content is 36% and has 88% of the particles under the size of 45 μm; the specific density is 2.80 g/cm^3^ [54,79].

Two types of fibers were used as reinforcement: green tow flax and carbon fibers [44]. They were added as 1% by mass of the composites. Green tow flax fibers are by-products of textile fiber production—they are coarse, broken fibers, removed during flax processing. After the process, they were dried. The fibers used for this study were shortened to around 5 mm in length. The fibers were delivered to the Faculty of Material Engineering and Physics, Cracow University of Technology by the Institute of Natural Fibers and Medicinal Plants (Poznan, Poland). Carbon fibers (P.P.H.U. SURFPOL Jacek Woźniak, Rawa Mazowiecka, Poland) have a length of 5 mm and a diameter of 8 μm [44].

### 4.2. Sample Preparation

Firstly, the sodium promoter was prepared by mixing a 10-molar (10 M) sodium hydroxide solution (NaOH) combined with a sodium water glass type R-145 solution (with molar module 2.5 and density about 1.45 g/cm^3^). The proportion 1:2 was used. The alkaline solution was prepared by pouring an aqueous solution of sodium silicate into flakes of technical sodium hydroxide dissolved in tap water. The solution was left until the concentrations equalized and ambient temperature was reached (around 2 h).

Next, the samples were prepared using sodium promoter, fly ash, sand and fibers (1% by mass). The solid ingredients were added first, and then the liquid one. The components were mixed to receive the homogeneous paste. The mixing time was about 10 min on a low-speed mixing machine. Then, two methods of production were applied for the sample manufacturing:

(1) “CAST”, in which traditional pouring molding was applied;
(2) “3D”, in which the sample was made by injection molding to simulate the 3D printing process.

Finally, all samples (casted and injected) were heated in the laboratory drying cabinet for 24 h at 75 °C. Afterwards, the samples were unmolded and stored in laboratory conditions (temperature ca. 20 °C, relative humidity ca. 50%). The four series of samples were prepared (Table 6).

### 4.3. Methods

The compressive strength test was carried out in accordance with the EN 12390-3 procedure described in the standard for concrete EN 12390-3 (“Testing hardened concrete. The compressive strength of the test specimens”) and the flexural strength test was carried out in accordance with the standard EN 12390-5 (“Testing hardened concrete and flexural strength of the test specimens”). Both tests were carried out on a Matest 3000 kN universal strength testing machine (Matest, Treviolo, Italy) with a speed of 0.05 MPa/s, calibration accuracy: class 1. For each analyzed composition of geopolymer composites, a minimum of four samples were involved. The cubic samples (compressive strength) have the dimensions: 50 mm × 50 mm × 50 mm, and the prismatic samples (flexural strength) have the dimensions: 50 mm × 50 mm × 200 mm, with the distance between the support points equal to 150 mm.

Analysis of bonding in the material was carried out by the Department of Medical Physics of the Jagiellonian University with the use fourier-transform infrared spectroscopy (FT-IR) Spektromer FT-IR Nicolet 6700 (Nicolet, Klapálkova, Czech Republic).

The chemical and mineralogical composition were investigated by the Department of Silicates and Macromolecular Compounds, Faculty of Materials Science and Ceramics, AGH University of Science and Technology, with the use of spectroscopy (X-ray diffraction and X-ray fluorescence). X-ray fluorescence (XRF) was performed on Spektrometr WDXRF Axios mAX, equipped with Rh source Rh 4kW (PANalytical, Malvern, UK). XRD was analyzed using an X’Pert Pro MPD diffractometer (PANalytical) with CuKα radiation at 30 mA and 40 kV. The 2θ angle varied between 20° and 53° with a step of 0.04° and with an accumulation time of 7 s for each step.

Nuclear magnetic resonance spectroscopy (NMR) was provided by Department of Magnetic Resonance Tomography, The Henryk Niewodniczański Institute of Nuclear Physics Polish Academy of Sciences. There were made on pulsed magnetic resonance spectrometer (MS NMR), with the following parameters: APOLLO spectrometric console by Tecmag (two broadband channels for the 5–450 MHz range, a digital receiver with a 14-bit ADC converter and a digital filter, a pulse recording programmer with a start of 100 ns), AMT M3426 broadband high-frequency power amplifier (1 kW power in the range 30–120 MHz), Usoft PA300 high frequency narrowband power amplifier (1kW power at 300 MHz), MAGNEX superconducting magnet (7 T magnetic field, 89 mm gap, field homogeneity 0.2 ppm for a 5 mm sphere), Bruker HP-WB 73A CP/MAS probe (two-channel, X channel: 44–122 MHz (2H-31P), 1H channel: 300 MHz, maximum spin frequency 15 kHz).

Research with the use of an atomic force microscope (AFM) was carried out by the Institute of Metallurgy and Materials Science of the Polish Academy of Sciences. The microscope ECSPM, Innova Bruker (Ettlingen, Germany), with NanoScope Analysis overview was provided. Bruker SPM Innova enables tests with tests up to 40 mm and thickness up to 18 mm in the range up to 100 μm for XY and up to 7.5 μm in Z in the contact and noncontact AFM modes. The apparatus also allows for atomic launch and allows for atomic thresholds in high-resolution mode. Image analysis software NanoScope Analysis aids correction and nonfogging results such as surface roughness, height of atomic thresholds, etc. NCHV measuring probes by Bruker on antimony-doped silicon with a sensor with the following characteristics were used: blade with radius R = 8 nm, beam stiffness: 42 N/m and resonance messages: approx. 330 kHz. The scanned area was selected each time for the possibilities for each sample. The method has a qualitative character.

The confocal microscopy investigations and scanning electron microscope (SEM) images were carried out in the Department of Material Science, Faculty of Mechanical Engineering, Technical University of Liberec. The SENSOFAR Metrology material confocal microscope working according to the ISO 25,178 standard was used for surface investigation on breakthroughs. Microstructure investigations with the use of the Carl Zeiss ULTRA Plus scanning electron microscope (SEM) type were also carried out on breakthroughs. The microscope has the following parameters: resolution: 1 nm @ 15 kV; 1.6 nm @ 1 kV, magnification: 12–1,000,000× in SE mode and acceleration voltage: 0.02–30 kV).

## 5. Conclusions

The investigation provided in the article focused on determining the influence of the microstructure of the material on the mechanical properties of the two kinds of geopolymer composites (flax and carbon reinforced) and the comparison of two methods of production for geopolymer composites (casting and 3D printing). By analyzing the results of the research presented in this paper, the following conclusions can be drawn:

(1) The best mechanical properties was reached for the sample made by simulating 3D printing process for the composite reinforced by flax fibers. It was 48.7 MPa for the compressive strength and 9.4 MPa for flexural strength.
(2) FT-IR clearly shows the presence of aluminum silicates and/or aluminosilicates in geopolymers. The analysis shows that all investigated geopolymers composites have a similar internal structure, consisting of free O–H, hydrogen bonded O–H, C–O, Si–O–Al and Si–O–Si as well as the crystalline phase of quartz.
(3) The main elements of the composite structure showed by XRF are: oxygen (O), silica (Si), and aluminum (Al). The main oxides presented in the structure showed by XRF are: alumina-Al_2_O_3_ and silica-SiO_2_. Moreover, the composite includes significant amounts of: Fe_2_O_3_, Na_2_O and CaO. The obtained data are coherent with the applied raw materials, particularly they confirmed the applied fly ash has a composition relevant for the class F. These elements come from raw materials used and are the basic element for creating the structure of the geopolymer and are similar for all investigated compositions.
(4) Identified phases showed by XRD are quartz, mullite, hematite, magnetite, anhydrite, albite, and sylimanite. A significant amount of quartz is associated with the use of sand as an aggregate. Among them, there are crystalline as well as amorphous phases. The mineralogical composition for all investigated materials is similar.
(5) NMR—The presented data show that all composites have a similar internal structure typical for geopolymer material. This investigation confirms, the presence of SiO_4_ and AlO_4_ tetrahedrons in a three-dimensional structure what is crucial for geopolymer structure.
(6) The AMF shows better coherence of the geopolymer made in additive technology to the reinforcement. The surface of the fibers is more evenly covered than in casted samples.
(7) The observations by confocal microscopy did not reveal significant differences between the samples produced by different methods. The fiber distribution is similar for all investigated compositions.
(8) The SEM observations show that the flax composites are characterized by better coherence between the fiber and the matrix. This partly explains the better mechanical properties of the samples with flax fiber. The microstructure observation does not explain the better results of compressive and flexural strength for the samples prepared by the additive technology method simulation (difference between production methods).

The results show the samples made by the additive technology had comparable properties with those made by casting method and similar structure. There is a lack of visible tendency to change the mechanical properties of materials depending on the manufacturing method used.

The application of additive technology in the construction industry could bring many advantages, such as low labor costs, less waste, and high efficiency. One of the main reasons for the limited use of this technology is the lack of material investigations. The materials still require development and optimization. One of the promising possibilities for further development is the reinforced geopolymer with fiber, which could be an eco-friendly alternative for concrete in many applications.

## Figures and Tables

**Table 1:** Types of fibers used in geopolymer composites manufactured by 3D printing.

Fiber	Matrix	Fiber Influence	Source
Steel micro-cable stainless steel grade SUS304; diameter: 1.2 mm; amount: 0.8% by vol.	Fly ash class F (0.64), silica fume (0.11), slag (0.25), fine silica sand (1.2), sodium metasilicate pentahydrate powder (0.125), tap water (0.348), PP fibers (0.0056) and VMA, hydroethylcellulose.	The 3D printed micro-cable reinforced geopolymer composite reaches the highest flexural strength (up to eight times) and deflection resistance (up to seventy times) when the filaments are deposited in an incline-crossed printing configuration compared to a nonreinforced one.	[30]
	Class F fly ash, ground granulated blast furnace slag, silica fume, sand; Sodium metasilicate pentahydrate powder	The failure mode of the reinforced structures changed from brittle to ductile and the microcable reinforcement altered the patterns of evolution of the strain. The reinforcement increases resistance to deformation and damage. The test results demonstrated that the micro-cables are conductive. Improved the load capacity of spiderweb-like structures by 132%. The bond between the geopolymer and cable reinforcement was proven to be effective.	[38]
Hooked-end steel fibers; 40 mm length; diameter 0.615 mm; 1 wt% Polypropylene fiber; length: 50 mm; 0.5 wt%	F fly ash and sand; sodium silicate and sodium hydroxide solution with a concentration of 8.0 M were utilized as activators	Inclusion fibers had negative effects on the bond strength between layers.	[33]
Steel microcable (diameter: 1.2 mm) Nylon microcable (1.3 mm) Carbon microcable (1.4 mm) Aramid microcable (0.8 × 1.2 mm) Polyethylene microcable (1.2 mm)	Class F low-calcium fly ash, ground granulated blast furnace slag, silica fume, sand with a maximum particle size of 1 mm; Penta sodium metasilicate powder	Stiffness of the cable is an important factor—nylon, carbon fiber, aramid, and polyethylene cables with stiffness less than that of steel cables are found to knot and are not suitable for embedding in printing filament. The tensile behavior depends on the cable reinforcement configurations. Polymer-based cables are better at increasing corrosion resistance than steel cables. The multi-cables introducing system is recommended for real construction practices.	[32]
Polyvinyl alcohol (PVA) fibers; length: 8 mm; amount 0.5 wt% Stainless steel cable-SUS304; varying diameter: 1, 1.5 and 2 mm.	80% class F grade fly ash (FA) 15% ground granulated blast furnace slag and 5% micro silica, fine river sand; potassium silicate	The steel cable could improve the flexural strength of 3D printed material by 290%.	[31]
Micro PVA fibers; diameter 26 μm, length: 6 mm	Fly ash and ground granulated blast furnace slag; anhydrous sodium metasilicate (solid activator)	Fibers maintain the mechanical performance and durability of the printed element.	[39]
PVA fibers; length: 3 mm, amount: 0.5 wt%	95% metakaolin and 5% silica fume; sodium silicate and 10 M sodium hydroxide solutions	Reduction in shrinkage in 3D printed, multifunctional geopolymer sensor—repair for concrete structures was presented.	[40]
Oil-coated PVA; diameter: 40 μm; length: 8 mm	Class F fly ash and granulated ground blast furnace slag; anhydrous sodium metasilicate powder with SiO_2_/Na_2_O mass ratio of 0.9	Increasing compressive strength, modulus of rupture, and deflection capacity. The orientation of the fibers in the 3D-printed samples was found to be mainly parallel to the printing direction. The inclusion of short polymeric fibers results in higher porosity due to the entrapment of air in the mixture.	[36]
PVA Polypropylene (PP) Polyphenylene benzobisoxazole (PBO) All 6 mm length	Class F fly ash, silica sands; sodium-based activator composed of 8.0 M NaOH and N Grade Na_2_SiO_3_ solutions	The flexural strength of the 3D printed fiber-reinforced geopolymer mixtures was substantially higher for all 3 types of fibers than that of the 3D printed geopolymer without fiber.	[35]
PP fibers; length: 6 mm, four different fiber contents were chosen: 0.25, 0.50, 0.75, and 1.00% by vol.	Fly ash, micron-scale silica sand; alkaline solution composed sodium silicate and sodium, and sodium carboxymethyl cellulose (CMC) powder were used	Shape relations ability improved with fiber content. Fibers increase the compressive strength of the material only in the perpendicular direction (parallel alignment of the fibers with the direction of the extrusion). Fiber increases the ductility, deflection capacity, and fracture energy. Increasing the volume of the fiber reduced the strength of the interlayer bond to some extent.	[37]
AR glass fibers (6 mm) Wollastonite (5–170 μm)	Metakaolins, calcined argillite, callovo-oxfordian argillites, kaolin, sand; potassium silicate	The addition of wollastonite or glass fibers increases the viscosity and decreases the workability (castability) of the material. The fibers are oriented parallel to the printing path during the process.	[41]
Short glass fiber; lengths: 3, 6 and 8 mm; amount: 0.25%–1% by vol.	Fly ash (class F), slag, micro silica, fine (river) sand; liquid potassium silicate; hydroxypropyl methylcellulose	The addition of fiber barely improves compressive strength and significantly flexural and tensile strength.	[34]
Green tow flax fibers; length 30–50 mm; amount 1 wt% Carbon fibers; length 5 mm, diameter of 8 μm; 1 wt%	Class F fly ash, sand; aqueous solution of sodium hydroxide with 10 M, and an aqueous solution of sodium silicate, in a ratio of 1:2.5	The inclusion of the fibers slightly enhanced compressive strength, and significantly enhanced flexural strength. The performance of samples containing flax fibers was better than that of samples containing carbon fibers.	[16]
Green tow flax fibers; length 30–50 mm; amount 1 wt%.	Class F fly ash, sand; aqueous solution of sodium hydroxide and sodium silicate	The results for compressive and flexural strength are better for plain samples than ones with fibers, regardless of the technology of samples’ manufacturing (3D printing and casting).	[42]

**Table 2:** Summarising the results for FT-IR for the geopolymer composites.

No	Main Peak	Maximum	Local Maximum	Bounding
1	3696–3132	3548	---	O-H
2	---	2931	---	O-H
3	---	2854	---	C-H
4	1754–1581	1654	---	H-O-H
5	1581–1357	1469	---	C-O
6	1357–821	1019	---	Si-O-Al or Si-O-Si
7	821–657	780	694	Si-O-Si
8	657–404	462	560	Si-O(Si)

**Table 3:** The elemental composition.

Element	Fly Ash	Sand	3DC	CASTC	3DF	CASTF
O	47.177	51.197	45.480	45.458	45.679	46.035
Si	22.693	40.902	25.435	25.575	25.889	26.645
Al	16.816	3.1760	9.7722	9.6359	9.8108	9.6718
Na	1.5975	0.86054	7.6957	7.2118	6.9284	6.5153
Fe	4.2531	1.0207	4.1249	4.4371	4.1543	3.9194
Ca	2.1660	0.89557	3.1752	3.3382	3.0991	3.0672
K	2.1515	1.3310	2.0509	2.1184	2.2392	2.0693
Mg	1.1472	0.23151	0.74137	0.72434	0.75079	0.74537
Ti	0.65890	0.15722	0.64831	0.65113	0.60643	0.57807
S	0.75313	0.036447	0.31958	0.27343	0.26437	0.23894
P	0.21729	0.035690	0.13598	0.11976	0.13932	0.12317
Ba	0.09688	0.037588	0.090645	0.11115	0.09866	0.085606
Mn	0.068042	0.015097	0.058950	0.078441	0.077765	0.059950
Cl	0.034838	0.012234	0.056550	0.059827	0.057638	0.046814
Sr	0.048700	0.017301	0.049155	0.051727	0.051172	0.045803
Cr	0.020727	0.060160	0.027897	0.030106	0.037666	0.040166
Zr	0.024358	0.014688	0.024719	0.030090	0.028394	0.025463
Nd	---	---	0.017149	---	---	---
Zn	0.023821	---	0.016475	0.022908	0.016359	0.022489
Cu	0.013335	---	0.016279	0.015339	0.015605	0.011612
Pb	0.014866	---	0.016108	0.013911	0.017128	0.014254
Ni	0.010272	---	0.014821	0.013401	0.012586	0.013197
Rb	0.013641	---	0.013645	0.015159	0.014523	0.013760
Co	---	---	---	0.015016	0.011711	0.011752

**Table 4:** The oxide composition.

Oxide	FLY ASH	Sand	3DC	CASTC	3DF	CASTF
Na_2_O	2.153	1.160	10.374	9.721	9.339	8.782
MgO	1.902	0.384	1.229	1.201	1.245	1.236
Al_2_O_3_	31.773	6.001	18.464	18.207	18.537	18.275
SiO_2_	48.548	87.502	54.453	54.713	55.386	57.003
P_2_O_5_	0.498	0.082	0.312	0.274	0.319	0.282
SO_3_	1.881	0.091	0.798	0.683	0.660	0.597
K_2_O	2.592	1.603	2.471	2.552	2.697	2.493
CaO	3.031	1.253	4.443	4.671	4.336	4.292
TiO_2_	1.099	0.262	1.081	1.086	1.012	0.964
Cr_2_O_3_	0.030	0.088	0.041	0.044	0.055	0.059
MnO	0.088	0.019	0.076	0.101	0.100	0.077
Fe_2_O_3_	6.081	1.459	5.898	6.344	5.939	5.604
NiO	0.013	---	0.019	0.017	0.016	0.017
CuO	0.017	---	0.020	0.019	0.020	0.015
ZnO	0.030	---	0.021	0.029	0.020	0.028
Rb_2_O	0.015	---	0.015	0.017	0.016	0.015
SrO	0.058	0.020	0.058	0.061	0.061	0.054
ZrO_2_	0.033	0.020	0.033	0.041	0.038	0.034
BaO	0.108	0.042	0.101	0.124	0.110	0.096
Nd_2_O_3_	---	---	0.020	---	---	---
PbO	0.016	---	0.017	0.015	0.018	0.015
Co_3_O_4_	---	---	---	0.020	0.016	0.016
Cl	0.035	0.012	0.057	0.060	0.058	0.047

**Table 5:** Spectrum analysis ^27^Al and ^29^Si MAS-NMR.

Sample	^27^Al MAS-NMR			^29^Si MAS-NMR		
	Position [ppm]	Width Half [ppm]	Relative Intensity [%]	Position [ppm]	Width Half [ppm]	Relative Intensity [%]
3DC	53.6 30.3 8.2	25.0 15.6 17.2	73 7 20	−94.0 −106.3	30.1 5.2	94 6
CASTC	54.0 29.9 8.2	25.8 16.1 18.5	75 6 19	−94.0 −105.3	30.9 3.8	98 2
CASTF	54.2 29.5 8.6	25.8 15.5 16.6	74 7 19	−93.6 −108.2	29.1 6.6	94 6
3DF	53.6 31.3 8.4	24.2 16.1 18.5	70 8 22	−93.3 −105.3	28.0 5.6	94 6

**Table 6:** Designation of manufactured composites.

**Designation**	**Mixture Proportion (% by Weight)**				**NaOH Solution**	**Production Method**
	**Fly Ash**	**Sand**	**Carbon Fiber**	**Flax Fiber**		
**3DC**	49.5	49.5	1.0	-	10 M sodium hydroxide solution + water glass (1200 mL in total)	injection molding to simulate 3D printing
**CASTC**	49.5	49.5	1.0	-		traditional molding (casted)
**3DF**	49.5	49.5	-	1.0		injection molding to simulate 3D printing
**CASTF**	49.5	49.5	-	1.0		traditional molding (casted)

## Data Availability

Not applicable.
